# The Public Health: Interviews with Local Authorities

**Published:** 1922-10

**Authors:** 


### THE PUBLIC HEALTH.

### INTERVIEWS WITH LOCAL AUTHORITIES.

### IV.—THE CITY OF SHEFFIELD.

IF, on a morning more fitting for November or February than early September, the visitor to Sheffield gets his first impressions through a wet, begrimed mist, across the dark waters of a canal, in the form of factories, chimneys and blackened buildings, he may say that here, indeed, is the "abomination of desolation." But he must wait and come to a more considered judgment. An expenditure of twopence will take him, by one of the tram routes, into delectable surroundings, not so easily attainable at so small an expenditure of time and money in many another of our great cities. Nay, if the sun and the wind get busy during the day, he shall stand in the heart of the city, and, a black cathedral and other black buildings notwithstanding, see real white clouds and real blue sky overhead. And if, in the meantime, he is so fortunate as to have been instructed at first hand in the way that those in authority are tackling the health problems of the city, he will begin to understand why, according to a recently published investigation, not one person in Sheffield out of ten was found who wished to live anywhere else.

Two obvious corrections must, for the same reason, be made at once in the visitor's point of view. Those chimneys belching forth black smoke in the early morning are not "tolerated." Whoever the responsible persons are, the Medical Officer of Health will get on their tracks ; many representations have been made already and prosecutions instituted in suitable cases. Thus, Sheffield is tending to become less black than she has been painted. The other correction is not a pleasing one. From beyond those fair stretches of country which gladden the eye at the end of the twopenny tram journey, impure and disease-carrying milk finds its way too often into Sheffield, so that, despite all the activities of Health Authorities, Dr. Wynne, the Medical Officer of Health, well-nigh despairs of seeing a constant supply of pure milk during the present generation, and recommends the use at all times of dried milk. Thus, the fair country side must have a black mark against it so long as so many of the farmers fail to realise the importance of care and cleanliness in the production of milk and its handling at all stages.

#### Dried Milk.

Councillor Kaye, the Chairman of the Public Health Committee, who, with Dr. Wynne, dealt kindly and patiently with our inquiries, gave it as his considered view that the use of dried milk was the only way of safety. He shares with Dr. Wynne the view that until those responsible for the milking of the cows can be educated to realise that the washing of the milkers' hands, the sterilisation of all utensils by steam, the cooling of the milk in separate clean chambers, are not the mere fads of an enthusiast, and that clean, well-lit cowsheds and yards are essential—all milk should be dried. In addition to the destruction of germs in the process of desiccation, the big economy in cost of carriage is important.

To the use of dried milk, as to certain other important factors, such as measures taken for the prevention of fly-breeding, is attributable, in part, the fact that in 1921, with a ground temperature the same as that experienced in 1911, the deaths in Sheffield from diarrhœa and enteritis under two years of age were 229 only (a rate of ·46 per 1,000), as compared with 527 (a rate of 1·16) in 1911.

#### Infant Welfare.

The last-mentioned fact must not be allowed to indicate that due tribute is not to be paid to the activities of lady inspectors, and to the Child Welfare work generally of the last few years. Here in Sheffield is to be observed the encouraging decline noted elsewhere in the infant mortality rates, a decline from an average figure of 160 in the five years 1902 to 1906 to just under 100 in the years 1919, 1920 and 1921.

The work in the Maternity and Child Welfare centres continues to increase, the attendances at baby consultations having grown from 39,316 in 1919 to 54,078 in 1921. Dried milk and other foods were supplied at cost price, during the financial year 1921-2, to the value of no less than £23,766. As regards free supply to necessitous persons, the Health Committee in Sheffield—with that combination of hard heads and soft hearts which Dr. Wynne notes as an essential feature of a good health administration—exercise a rigid scrutiny over all claims, and have to record a current expenditure of less than £500 a year in this direction. Not the least remarkable feature of this expenditure, abnormally low though it is, compared with other areas, is that it has been concurrent with a low infant mortality rate—this notwithstanding, also, the serious unemployment in the city.

#### Infectious Diseases.

There is, in Sheffield as elsewhere, a virtual disappearance of small pox and typhoid fever, and a decline, not observed to the same extent as elsewhere, in the death rate from diphtheria, the figure of ·04 per 1,000 of the population being the lowest recorded for this disease in the city. There is the same failure on the part of parents, here as elsewhere, to appreciate the fact that measles must be treated more seriously, although in this matter, with the effective co-operation of the Fever Hospital Superintendent, and a readiness on the part of parents to send their children into hospital, the epidemic of 1922 was effectively checked.

In regard to the treatment at venereal clinics, we think it well to stress the view expressed elsewhere by Dr. Wynne that those who can afford to do so should pay for their treatment.

#### The Treatment of Tuberculosis.

The tuberculosis problem, as it is viewed in Sheffield, is that of the expenditure of a limited sum to the best advantage, particularly so far as regards residential treatment. Early cases are by no means neglected, specially where children are concerned. "Middle" cases are treated mainly at the dispensary. But it is the isolation of the advanced cases, for as long as possible, to which the efforts of the Public Health Committee and their Medical Officer are directed, that thereby the greatest good of the greatest number may be served. Dr. Wynne and his colleague, Dr. Rennie, in their recommendations to the Committee, are thus seeking to avoid, to some extent, that " wrong turning in public expenditure " with which the former deals in the paper quoted at the end of this article. The restoration of the working capacity of a selected individual is, to put it plainly, a relatively small matter compared with the avoidance of the infection of several individuals to the undoing of their working capacity, and the consequent avoidance of the later infection by them of their associates.

In passing, it may be observed that the disparity, already noted in the article on Birmingham, in the morbidity rates for tuberculosis amongst males and females, respectively, is greater in Sheffield, the proportions of deaths, males to females, over a period of 10 years being as 16 : 9.

*Mortality amongst Grinders.*—Pulmonary tuberculosis takes heavy toll amongst the grinders. From a return furnished for the years 1901 to 1910, it would appear that some 40,000, nearly one-third, of the males of 18 years and over were engaged in the grinding trade and its branches ; and the grim fact has to be recorded that, in the five years 1908-12, no less than 51 per cent. of the deaths among grinders from all causes were attributable to pulmonary tuberculosis. The percentage has fallen to 36 in the year 1921, and it is noteworthy that this decline is concurrent with the operation of more stringent regulations affecting the conditions under which the grinders carry on their work.

Mortality due to this cause is also very high amongst ganister workers. There are miles of workings of ganister seams around Sheffield. Ganister is used largely in making the linings of furnaces.

### Housing—Possibilities of Hut Conversion.

It would be wearisome, perhaps unprofitable, to relate in detail the story of the housing deficiency in every area we visit. In County and in County Borough alike, the same sad tale persists. The overcrowding in Sheffield is as bad as, or worse than, at any time in its history. And it is not a problem of housing strangers to Sheffield, drawn in during the manufacture of munitions through the period of war, and now staying on hoping for something to " turn up." If this is a contributory cause, it is only so to a very small extent. It is the housing of the normal workers in Sheffield industries, vast numbers of them now out of employment, that is the main problem. The Housing and Town Planning Act of 1909 gives the necessary authority for reconstruction of slum areas on a sane and sound plan. But who is to provide the means ? With the stoppage of Government subsidies—themselves an incentive to waste and extravagance, interpolates Dr. Wynne—with rates at more than 20s. in the £, with, in addition, resort to loans for current poor law relief, with the foreign markets for all Sheffield's wares waiting, but unable to pay for them, so that here, and there, and there again, the machine stops running, who is going to pay ? It must not be inferred that nothing is being done, but it is all too little. One interesting possibility was mentioned, namely, that the Air Ministry huts in a camp outside the town should undergo conversion, and be used for " accommodation " houses, to which the dwellers in overcrowded areas where houses are crying out for reconstruction might migrate while the work of rebuilding is going on. Before the Council, however, could embark on the necessary outlay on acquiring and converting the huts, they would need to be reasonably sure, in the first place that they could get the people to go into them, and in the second place that they could get them out again. As to the first difficulty, there is apparently a reluctance on the part of the Sheffield worker to go to a distance—even the length of a twopenny tram ride—from his work.

Our attention was drawn to a fact, noticeable elsewhere, that another Act of Parliament of 1909-10 rendered the private builder very shy of putting up new houses and prevented the beneficent activities contemplated by the Housing and Town Planning Act of 1909 from having full play. If we look at the figures for new houses certified from 1908 onwards, we shall see that the housing arrears were piling up for years before the war, and that the post-war problem has thereby been rendered more acute. The numbers each year, from 1908 to 1914, are : 1,778, 1,469, 1,243, 866, 703, 542, 570.

When Dr. Wynne is most depressed after a visit to insanitary areas, he turns for comfort to a report in his possession on the condition of Sheffield in 1846. Things have moved along, although there is still a long and difficult road to negotiate. This report, he says, gives in elaborate detail the condition of yards ankle-deep in putrefying ordure from privy-middens used by from fifteen to twenty families. In this the children played and the liquid material drained into the houses. There is one case of a privy serving nineteen families and the children attending a *school built over it*, a frequent arrangement in those days. Everywhere, in the poorer parts of the town, there were rotting piles of vegetable and other refuse unremoved except by the natural processes of decay. The water supplied to the town in those days is described as being of good quality, but " the supply of water in many parts of the town is not kept up for more than two hours at a time, twice or three times a week, and this being distributed to the humbler classes by means of public stand-pipes, many find great difficulty in procuring a sufficient quantity for ordinary domestic purposes, much less for scouring their houses and courts. Many persons are in the habit of storing the water in wooden tubs placed before their doors, when it soon becomes foul from the deposit of soot and other foreign matters so as to be unfit for use." The well water is described as being freely polluted by putrid material from the conditions enumerated in the first part of the report. Ventilation of the houses was " by apertures in doors and windows," and even these had to be kept closed owing to the abominable stench from outside. Ventilation of schools, workshops, and public buildings was quite simply ignored. It is not surprising that in 1832 Sheffield was visited by an epidemic of cholera which caused over 400 deaths in six months, and that the normal death rate of the town at the time of this report was *over* 28 *per* 1,000.

The City Council of Sheffield believe that they have in their present Medical Officer "the right man," whose practical sense of administrative efficiency is not blurred by foolish sentiment or a distorted "vision." For his part, we venture from personal observation to say that Dr. Wynne finds encouragement in the support which he receives from a Committee presided over by so kindly, observant, and "hard-headed" a public servant as Councillor Kaye.

(*We hope in another issue to make reference to the interesting Sheffield Sewage Disposal Scheme under the management of Mr. Haworth.*—Ed.)

---

In the course of our visit to Sheffield, we were fortunate in having from Dr. Wynne an expression of his views in amplification of the interesting paper, read by him at the British Medical Association meeting at Glasgow, in July, on "The Wrong Turning in Public Health," from which we print a considerable extract. This article appeared in the "British Medical Journal" of August 26.

Dr. Wynne disclaims any cynical attitude towards public expenditure on the individual. He would desire that all such existing expenditure should be wisely directed, and that any further increase in the cost of services on the individual, rather than on his environment, should be closely scrutinised before being embodied in hasty legislation. He is strongly opposed to public expenditure on those individuals who can afford to pay for treatment, and cites some of the expenditure at the venereal clinics as a disturbing example of this defect in administration. And, very much alive as he is to the need for the most rigid economy in all forms of public expenditure at the present time, Dr. Wynne wants to see every *further* pound that can be spared devoted to prevention, to improvement of the environment. In an arresting statement, after paying a high tribute to the work of the child welfare centres in the country generally, he says: "Mere saving of child life is not enough; we must make these lives worth living. The very fact that we are causing so many infants to survive seems to me only to emphasise the duty of providing them with an environment in which they can be happy."

The tendency of the developments in recent years on public expenditure on the diseased individual is creditable to our consciences, and to the emotional side of our natures. But it is essential to bid the enthusiast and the sentimentalist remember, says Dr. Wynne, that poverty is the principal cause of public ill-health, and that any expenditure which is not justified by actual results increases poverty, and therefore the incidence of disease and mortality.

Asked as to his views on the medical provisions of the National Health Insurance Scheme, Dr. Wynne stated that he considered that its basic principle was right. Under this scheme it is directly in the doctor's interest to get a patient well, and to keep him well. But there are too many insured persons crowding the doctor's surgeries for trifling ailments; they are not being educated away from the fetish of the 8-oz. bottle of medicine. To Dr. Wynne, an unchecked resort to this kind of bottle is fraught with trouble comparable with that which arises from a too-frequent application to one of another kind, where the disturbing effect on the individual, and on the community, are more immediately manifest.

As to his views on the expenditure on tuberculosis, and on the medical inspection and treatment of school children, in pursuance of his thesis that disease must be attacked at its origin, and that expenditure on environment promotes the health of the mass of the people rather than that of selected individuals, the subjoined extracts from Dr. Wynne's most interesting article will speak for themselves.

